# Effects of dietary mineral manipulation on productive performance and eggshell quality in laying hens during the late laying period

**DOI:** 10.1016/j.psj.2026.107880

**Published:** 2026-10-05

**Authors:** Shahab Pourlorestani, Mehran Torki, Reza Mahdavi

**Affiliations:** Animal Science Department, College of Agriculture and Natural Resources, Razi University, Kermanshah, 6715685423, Iran

**Keywords:** Layers, Eggshell, Magnesium, Manganese, Zinc

## Abstract

This study evaluated mineral-based dietary interventions as a strategy to mitigate the age-related decline in egg production and quality commonly observed in late-cycle Lohmann Selected Leghorn laying hens. A completely randomized design was used with 294 hens at 90 wk of age assigned to 7 dietary treatments for 56 d:(1) basal control diet; (2) basal diet supplemented with 4 g/kg MgO; (3) basal diet supplemented with 50 mg/kg MnSO_4_; (4) basal diet supplemented with 30 mg/kg ZnSO_4_; (5) basal diet supplemented with MgO, MnSO_4_, and ZnSO_4_ at the respective levels; (6) basal diet containing 25% less available P than the control; and (7) basal diet containing 15% more Ca than the control.

Supplementation with Mg or Mn increased feed intake, whereas the combined Mg + Mn + Zn treatment reduced feed consumption (P ≤ 0.05). All dietary treatments improved feed conversion ratio throughout the experimental period, and increased egg production during 94–98 wk of age (P ≤ 0.05), with no effect on egg mass (P > 0.05). The high-Ca diet increased specific gravity and shell thickness, Mg supplementation increased shell weight, and Mn supplementation enhanced shell thickness (P ≤ 0.05). Zinc supplementation, alone or in combination with Mg and Mn, reduced the incidence of damaged eggs (P ≤ 0.05).Dietary treatments had no significant effect on internal egg quality traits (P > 0.05), with the sole exception of Haugh unit values, which were significantly higher in hens supplied the high-Ca diet (P ≤ 0.05). Overall, targeted mineral interventions, particularly increased dietary Ca and supplementation with Mg, Mn, or Zn, improved selected production and eggshell quality traits in aged laying hens.

## Introduction

The extension of laying cycles beyond 80 weeks of age has become increasingly common in commercial egg production (**EP**), creating nutritional challenges associated with aging hens. Advancing age is associated with reduced laying rate and egg mass (**EM**), deterioration of eggshell thickness and strength, and increased incidence of cracked eggs, partly because of changes in mineral metabolism, calcium (**Ca**) homeostasis, and skeletal mineral mobilization (Wistedt et al., 2019; Gu et al., 2021; Hou et al., 2025; Rezaei and Mens, 2024). These age-related changes highlight the importance of optimizing mineral nutrition in hens subjected to extended laying cycles.

Calcium and phosphorus (**P**) are primary drivers of skeletal integrity and eggshell formation (Li et al., 2017). Although Ca comprises 94–97% of shell mineral content, age-related declines in intestinal Ca absorption limit shell quality; conversely, excessive dietary Ca depresses FI and overall mineral utilization (Safaa et al., 2008; Diana et al., 2021; Jiang et al., 2023; Sinclair-Black et al., 2023). Similarly, while P is vital for energy metabolism, excess dietary P impairs Ca availability and elevates environmental excretion (Sinclair-Black et al., 2023). Recent findings indicate that modern laying hen P requirements are lower than traditional standards, with 2.2 g/kg non-phytate P (**NPP**) maintaining production and shell quality in older flocks (Rodehutscord et al., 2023; Pagnussatt et al., 2025; Shao et al., 2025).

In addition to macrominerals, trace elements, specifically magnesium (**Mg**), manganese (**Mn**), and zinc (**Zn**), act as essential enzymatic cofactors in matrix synthesis, bone metabolism, and shell calcification (Shastak and Rodehutscord, 2015; Zhang et al., 2017a; Elnesr et al., 2024). Given age-related decreases in nutrient utilization, targeted trace mineral fortification may benefit older hens, though reported responses remain variable (Skrivan et al., 2016; Wistedt et al., 2019; Ogbuewu and Mbajiorgu, 2022; Zarghi et al., 2023).

Despite extensive research on mineral nutrition, evidence remains limited for laying hens beyond 90 weeks of age, particularly regarding the comparative efficacy of different mineral strategies. Most previous studies have focused on individual mineral interventions, making direct comparisons among increased Ca, reduced available P, and supplementation with Mg, Mn, or Zn difficult. Therefore, the present study evaluated these contrasting strategies within a single experimental model to compare their effects on productive performance and egg quality in late-laying hens.

## Materials and methods

### Animals, housing, and management

All animal-related procedures were conducted in accordance with the ethical standards and guidelines approved by Razi University, Kermanshah, Iran, and applicable national regulations.

A 56-d feeding trial was conducted using 294 Lohmann Selected Leghorn (**LSL**) laying hens at 90 wk of age obtained from a commercial EP facility (Morghabi Company, Tehran, Iran). Before the experiment, the hens had been reared and managed according to standard commercial recommendations for Lohmann laying hens (Lohmann Tierzucht, 2020) under a single-stage, nonmolted production system. During the pullet and laying phases, the flock received standard commercial phase-fed mash diets formulated to meet or exceed the nutrient recommendations for LSL laying hens (Lohmann Tierzucht, 2020). The flock achieved a peak EP rate exceeding 92% and had an average EP rate of approximately 67% immediately before enrollment in the experiment.

Before initiation of the experimental treatments, all hens underwent a 30-d adaptation period during which they received the basal diet under uniform management conditions. At the end of the adaptation period, individual body weight and EP were recorded. These measurements were used to balance the allocation of hens across dietary treatments so that treatment groups had comparable initial body weight and laying performance.

The hens were housed in 98 wire cages, with 3 hens per cage (50 × 50 × 50 cm). Throughout the experimental period, ambient temperature was maintained at 20 to 22°C, and a lighting schedule of 15 h light and 9 h dark (15L:9D) was provided. Feed was offered daily at a restricted allowance of 120 g/hen/d using trough feeders accessible to all hens within an experimental unit. Fresh water was provided *ad libitum* through trough drinkers.

### Experimental design and dietary treatments

The experiment was conducted as a completely randomized design (**CRD**) with 7 dietary treatments and 7 experimental replicates per treatment. Two adjacent cages containing 6 hens in total (3 hens per cage) were defined as one experimental unit. Accordingly, the 294 hens comprised 49 experimental units. Cages were randomly assigned to treatments using a random number generator to ensure unbiased allocation. The use of seven replicates per treatment was based on power calculations from previous studies and provided at least 80% statistical power to detect differences of approximately 5–7% in EP at α = 0.05.

The basal diet was a conventional corn–soybean meal diet formulated for late-laying hens (90–98 weeks of age) and contained 2,600 kcal/kg metabolizable energy (**ME**), 14.2% crude protein, 3.98% Ca, and 0.32% available P (Table 1). Based on the mineral contributions of the feed ingredients and vitamin–mineral premix, the calculated basal diet provided approximately 1.60 g Mg/kg, 119 mg Mn/kg, and 89 mg Zn/kg. The calculated Mn and Zn concentrations were consistent with the recommended inclusion levels of 100 mg Mn/kg and 60 mg Zn/kg through the layer premix, as specified in the Lohmann Management Guide (Lohmann Tierzucht, 2020). Accordingly, the basal diet was formulated to provide mineral concentrations above minimum requirements, reflecting commercial safety margins to account for variability in ingredient composition and mineral bioavailability, as well as potential age-related declines in mineral absorption efficiency in hens beyond 90 weeks of age.

**Table 1:** Composition of experimental diets.

Feed ingredient (kg)	Control	25% less phosphorus than recommended	15% more calcium than recommended
Corn	60.73	60.69	61.46
Soybean meal	19.49	19.82	21.32
Oil	0.30	0.30	0.30
Wheat bran	7.25	7.43	3.01
Limestone (shell carbonate)	9.62	9.72	11.29
Dicalcium phosphate	1.50	0.93	1.51
Methionine	0.17	0.17	0.18
Salt	0.16	0.17	0.18
Sodium bicarbonate	0.28	0.27	0.26
Mineral premix	0.25	0.25	0.25
Vitamin premix	0.25	0.25	0.25
Calculated nutrients			
Metabolizable energy (kcal/kg)	2600	2600	2600
Crude protein (%)	14.20	14.28	14.33
Digestible lysine (%)	0.62	0.63	0.65
Digestible methionine + cysteine (%)	0.58	0.58	0.59
Digestible threonine (%)	0.45	0.45	0.46
Calcium (%)	3.98	3.92	4.59
Available phosphorus (%)	0.32	0.24	0.32

Vitamin–mineral premix supplied per kilogram of diet: Vitamin A 10,000 IU, Vitamin D3 2,500 IU, Vitamin E 20 mg, nicotinic acid 30 mg, cobalamin 25 mg, calcium pantothenate 10 mg, Vitamin K3 3 mg, thiamine 1 mg, riboflavin 4 mg, pyridoxine 3 mg, folic acid 0.5 mg, biotin 0.5 mg, choline chloride 400 mg, iron 25 mg (sulfate), copper 5 mg (sulfate), manganese 100 mg (sulfate), zinc 60 mg (oxide), iodine 0.5 mg (calcium iodate), and selenium 0.2 mg.

The seven experimental diets were as follows: (1) basal diet (control); (2) basal diet supplemented with 4 g/kg MgO; (3) basal diet supplemented with 50 mg/kg MnSO_4_; (4) basal diet supplemented with 30 mg/kg ZnSO_4_; (5) basal diet supplemented with MgO, MnSO_4_, and ZnSO_4_ at the respective levels described above; (6) a low-available-P diet containing 0.24% available P, representing a 25% reduction relative to the control diet; and (7) a high-Ca diet containing 4.59% Ca, representing a 15.3% increase relative to the control diet.

The mineral supplements were provided as MgO (43% Mg), MnSO_4_ (32% Mn), and ZnSO_4_ (34% Zn). Accordingly, inclusion of 4.0 g/kg MgO supplied 1.72 g supplemental Mg/kg diet, whereas inclusion of 50 and 30 mg/kg MnSO_4_ and ZnSO_4_ supplied 16 and 10.2 mg supplemental Mn and Zn/kg diet, respectively. Thus, the calculated total concentrations in the respective supplemented diets were approximately 3.32 g Mg/kg, 135 mg Mn/kg, and 99.2 mg Zn/kg. The combined Mg + Mn + Zn treatment received the same inclusion rates of the three mineral sources and therefore had the same calculated total concentrations of these minerals.

The low-P diet was formulated by reducing dicalcium phosphate from 1.50 to 0.93 kg/100 kg diet, resulting in a reduction in calculated available P from 0.32 to 0.24%. The high-Ca diet was formulated by increasing limestone from 9.62 to 11.29 kg/100 kg diet, resulting in an increase in calculated Ca concentration from 3.98 to 4.59%. For the mineral-supplemented diets, the specified amounts of MgO, MnSO_4_, and ZnSO_4_ were incorporated into the basal formulation, with proportional adjustments to the other basal ingredients to maintain a constant final diet weight. The complete ingredient composition and calculated nutrient concentrations of the experimental diets are presented in Table 1.

The chemical composition of feed ingredients (corn, soybean meal) was determined by laboratory analysis for crude protein (Kjeldahl method), ether extract (Soxhlet extraction), crude fiber, and ash, Ca (atomic absorption spectrophotometry), and total P (spectrophotometric molybdate-vanadate method). Analyzed values were used in diet formulation where available. Dietary formulation targets were based on the Lohmann LSL Commercial Management Guide (Lohmann Tierzucht, 2020). For nutrients not analyzed, ME was calculated using NRC (1994) prediction equations based on proximate composition. Amino acid composition was obtained from NRC (1994), and digestible amino acids were calculated using the corresponding true digestibility coefficients reported by NRC (1994). Total P was based on analyzed values, whereas available P was calculated using reference availability values from NRC (1994), assuming 30% phytate-P availability in the absence of phytase supplementation.

Diets were manufactured at the Razi University Feed Mill, Kermanshah, Iran. All ingredients were weighed according to their respective formulations using a calibrated electronic balance (±1 g accuracy). Before incorporation into the complete diets, each mineral supplement was premixed with approximately 5 kg of the basal diet to facilitate uniform distribution and minimize segregation. The premixes were then incorporated into the remaining ingredients and mixed in a vertical mixer for 10 minutes using the same procedure for all treatments to ensure uniformity and homogeneity. Diets were prepared as mash and stored in sealed containers at ambient temperature until use.

### Performance traits

Productive performance was evaluated throughout the 56-d experimental period. Eggs were collected daily from each experimental replicate and recorded for total egg number, egg weight (**EW**), and defective eggs (cracked or shell-less eggs). Feed refusals were collected and weighed weekly for each replicate. Feed intake (**FI**, g/hen/d) was calculated from the amount of feed offered (120 g/hen/d) and the corresponding feed refusal, adjusted for the number of live hens and days in each measurement period. Hen-day EP (%) was calculated as the number of eggs produced divided by the number of live hens during the corresponding observation period × 100. Mean EW was calculated as the total weight of eggs collected divided by the total number of eggs. Egg mass (g/hen/d) was calculated as EP (expressed as a proportion) × mean EW. Feed conversion ratio (**FCR**) was calculated as FI divided by EM.

### Egg quality traits

To evaluate internal and external egg quality, 2 eggs were randomly collected from each experimental replicate on d 28 and d 56 of the experimental period, resulting in 14 eggs per treatment at each sampling time. Prior to processing, individual EW was recorded using a precision electronic balance (±0.01 g; Shimadzu Corporation, Kyoto, Japan). Each egg was then broken onto a level glass surface. Albumen height (H) was quantified utilizing a tripod micrometer (Mitutoyo Corporation, Kawasaki, Japan; accuracy: 0.01 mm). The Haugh unit was subsequently computed using the standard formula: Haugh unit = 100 × log (H + 7.57 - 1.7W^0.37), where H denotes the albumen height (mm) and W represents the EW (g).

Following the separation of the egg components, the yolk was carefully cleared of any remaining albumen residues and weighed on the aforementioned electronic balance. Yolk quality was further evaluated via the yolk index, determined as follows: yolk index = (H/D) x 100, where H is the yolk height measured with the Mitutoyo tripod micrometer (accuracy: 0.01 mm), and D is the yolk diameter measured using a compass (Swordfish, China; accuracy: 0.02 mm).

Eggshell quality metrics included shell weight, thickness, and specific gravity. The broken shells were rinsed thoroughly under running water to eliminate adhering membranes and remnants, air-dried at room temperature for 24 hours, and weighed. Shell thickness was determined with a digital micrometer (Mitutoyo Corporation, Kawasaki, Japan) by averaging measurements taken at three distinct anatomical regions: the blunt end, the equatorial zone, and the sharp end. Finally, egg specific gravity was evaluated via the saline flotation technique. Eggs were placed sequentially into a series of sodium chloride solutions with densities increasing from 1.060 to 1.100 g/cm3 in 0.005 g/cm3 increments. The specific gravity was defined as the density of the specific brine solution in which the egg initially floated.

### Statistical analysis

Data were analyzed using the GLM procedure of SAS (version 9.1; SAS Institute, 2015). The experimental unit for all analyses was the 2-cage group containing 6 hens. Accordingly, productive-performance data were first summarized within each experimental replicate and analyzed by dietary treatment for the 90 to 94 wk period, the 94 to 98 wk period, and the overall 56-d experimental period.

Egg-quality measurements obtained on d 28 and d 56 were analyzed separately. Two eggs were collected from each experimental replicate at each sampling time, and measurements from the 2 eggs were averaged to obtain a single replicate value before statistical analysis. Thus, individual eggs were treated as subsamples rather than independent experimental observations.

Initial EP was used to balance treatment allocation among groups and was not included as a covariate. When a significant treatment effect was detected, means were separated using Tukey's honestly significant difference test. Statistical significance was declared at P ≤ 0.05.

The underlying statistical framework is defined below:

$$X_{ij}=\mu +T_{i}+e_{ij}$$

Where: $X_{ij}$ = the measured value of a given observation; μ = the overall population mean; $T_{i}$ = the fixed contribution of the dietary treatment (i = 1, 2, …7); $e_{ij}$ = the random experimental error.

## Result

### Productive performance

Table 2 outlines the outcomes of the various mineral supplementation strategies on the FI and EP of laying hens from 90 to 98 weeks of age. Feed intake was significantly influenced by dietary treatments during both experimental phases (90–94 and 94–98 weeks) as well as over the overall period (P < 0.0001). During 90–94 weeks, hens supplemented with Mg and Mn exhibited the highest FI values (111.91 and 111.95 g/day, respectively), which were significantly greater than other treatments. Similar trends were observed during 94–98 weeks, where Mg-supplemented hens maintained the highest FI (112.55 g/day), followed by Mn supplementation (111.77 g/day). In contrast, hens receiving the combined Mg + Mn + Zn supplementation showed the lowest FI during this period (109.86 g/day). Across the entire experimental period, Mg supplementation resulted in the greatest overall FI (112.23 g/day), followed by Mn supplementation (111.86 g/day), whereas the combined mineral supplementation treatment produced the lowest average FI (110.32 g/day).

**Table 2:** Effect of different mineral supplementation on feed intake and egg production of aged laying hens.

	Feed intake (g/hen/day)				Egg production (%)		
	90-94 wk		94-98 wk	Overall	90-94 wk	94-98 wk	overall
BD		110.56ᶜᵈ	111.04ᶜ	110.76ᶜ	62.1	51.40ᵇ	57.00
BD + Mg		111.91ᵃ	112.55ᵃ	112.23ᵃ	61.5	59.50ᵃ	60.70
BD + Mn		111.95ᵃ	111.77ᵇ	111.86ᵇ	67.0	53.40ᵇ	60.20
BD + Zn		110.88ᵇ	110.59ᵈ	110.74ᶜ	71.5	60.50ᵃ	66.00
BD + Mg + Mn + Zn		110.79ᵇᶜ	109.86ᵉ	110.32ᵈ	68.0	59.20ᵃ	63.50
BD - P		110.48ᵈ	111.08ᶜ	110.78ᶜ	65.4	59.50ᵃ	62.70
BD + Ca		110.58ᶜᵈ	110.87ᶜ	110.72ᶜ	67.8	58.80ᵃ	63.20
SEM		0.09	0.08	0.05	0.27	0.01	0.02
P-value		<0.0001	<0.0001	<0.0001	0.14	0.005	0.09

BD = basal diet (control); BD + Mg = basal diet supplemented with magnesium; BD + Mn = basal diet supplemented with manganese; BD + Zn = basal diet supplemented with zinc; BD + Mg + Mn + Zn = basal diet supplemented with magnesium, manganese, and zinc; BD−P = basal diet with reduced available phosphorus (75% of the basal diet level); BD + Ca = basal diet containing 4.59% Ca, representing a 15.3% increase relative to the basal diet.

SEM: Standard error of the mean.

Values within the same column bearing different superscripts (a–e) differ significantly (P ≤ 0.05).

Dietary treatments had no significant effect on EP during either the initial phase (90–94 weeks of age) or the overall experimental period. During 94–98 weeks, dietary treatments significantly affected EP (P = 0.005). Hens receiving Zn supplementation achieved the highest EP (60.5%), which was statistically similar to those fed Mg supplementation (59.5%), the combined Mg + Mn + Zn supplementation (59.2%), the reduced available P diet (59.5%), and the high-Ca diet (58.8%). These treatments significantly outperformed the control (51.4%) and Mn-supplemented groups (53.4%).

The effects of distinct mineral treatments on laying hen EM and FCR over the late laying cycle are presented in Table 3.Dietary modifications exerted no significant influence on EM during weeks 90–94, weeks 94–98, or across the entire study timeline (P > 0.05). In contrast, dietary treatments significantly influenced FCR during both experimental phases and over the overall period (P < 0.05). During 90–94 weeks, the control and Mg-supplemented groups exhibited poorer feed efficiency, with FCR values of 2.74 and 2.93, respectively, compared with all other treatments (P = 0.004). During 94–98 weeks, the control group again showed a significantly higher FCR (3.76) than all supplemented treatments, indicating reduced feed efficiency (P = 0.004). Considering the overall experimental period, the control group recorded the poorest FCR (3.25), significantly higher than all other treatments (P = 0.03).

**Table 3:** Effect of different mineral supplementation on egg mass and feed conversion ratio of aged laying hens.

	Egg mass (g/hen/day)				Feed conversion ratio (g/g)		
	90-94 wk		94-98 wk	Overall	90-94 wk	94-98 wk	overall
BD	40.09	30.46		35.42	2.74ᵃᵇ	3.76ᵃ	3.25ᵃ
BD + Mg	38.44	36.48		37.28	2.93ᵃ	2.98ᵇ	2.96ᵇ
BD + Mn	43.30	37.64		40.42	2.68ᵇ	3.09ᵇ	2.89ᵇ
BD + Zn	45.72	38.45		42.00	2.51ᵇ	3.05ᵇ	2.78ᵇ
BD + Mg + Mn + Zn	43.51	37.28		40.57	2.53ᵇ	3.05ᵇ	2.79ᵇ
BD - P	41.80	38.74		40.28	2.60ᵇ	3.01ᵇ	2.80ᵇ
BD + Ca	45.14	36.33		40.57	2.55ᵇ	3.11ᵇ	2.83ᵇ
SEM	1.82	2.41		1.89	0.07	0.14	0.10
P-value	0.07	0.25		0.21	0.004	0.004	0.03

BD = basal diet (control); BD + Mg = basal diet supplemented with magnesium; BD + Mn = basal diet supplemented with manganese; BD + Zn = basal diet supplemented with zinc; BD + Mg+ Mn+ Zn = basal diet supplemented with magnesium, manganese, and zinc; BD − P = basal diet with reduced available phosphorus (75% of the basal diet level); BD + Ca = basal diet containing 4.59% Ca, representing a 15.3% increase relative to the basal diet.

SEM: Standard error of the mean.

Values within the same column bearing different superscripts (a–e) differ significantly (P ≤ 0.05).

Table 4 illustrates how EW and the occurrence of broken or cracked eggs responded to the different nutritional strategies. Statistically significant treatment effects on EW were confirmed during both analytical intervals as well as the collective trial period (P = 0.005, P = 0.03, and P = 0.04, respectively). During 90–94 weeks of age, hens fed the diet containing 30% higher Ca than the recommended requirement produced the heaviest eggs (66.19 g), significantly exceeding all other treatments. The lowest EW during this period was observed in the Mg-supplemented group (63.68 g). Similar results were obtained during 94–98 weeks, where the high-Ca treatment again resulted in the greatest EW (65.10 g), although it was statistically similar to the Mg- and Mn-supplemented groups. Across the entire experimental period, hens receiving the high-Ca diet maintained the highest average EW (65.64 g), while the control group produced the lightest eggs (63.88 g).

**Table 4:** Effect of different mineral supplementation on egg weight and incidence of abnormal eggs of aged laying hens.

	Egg weight (g)			Damaged eggs (%)		
	90-94 wk	94-98 wk	Overall	90-94 wk	94-98 wk	overall
BD	64.34ᵇᶜ	63.42ᵇ	63.88ᵇ	10.95	11.82ᵃ	11.09ᵃᵇ
BD + Mg	63.68ᶜ	64.86ᵃ	64.27ᵇ	8.50	5.75ᵇ	7.51ᵃᵇᶜ
BD + Mn	64.97ᵇᶜ	64.58ᵃᵇ	64.77ᵃᵇ	12.46	11.31ᵃ	11.74ᵃ
BD + Zn	64.99ᵇ	64.48ᵃᵇ	64.74ᵃᵇ	6.78	4.22ᵇ	5.99ᶜ
BD + Mg +Mn+Zn	64.88ᵇᶜ	63.38ᵇ	64.13ᵇ	5.11	4.99ᵇ	4.79ᶜ
BD-P	64.37ᵇᶜ	63.95ᵃᵇ	64.16ᵇ	8.38	6.46ᵇ	7.59ᵃᵇᶜ
BD+Ca	66.19ᵃ	65.10ᵃ	65.64ᵃ	7.33	5.36ᵇ	6.48ᵇᶜ
SEM	0.40	0.43	0.38	1.89	1.61	1.57
P-value	0.005	0.03	0.04	0.13	0.004	0.02

BD = basal diet (control); BD + Mg = basal diet supplemented with magnesium; BD + Mn = basal diet supplemented with manganese; BD + Zn = basal diet supplemented with zinc; BD + Mg+ Mn+ Zn = basal diet supplemented with magnesium, manganese, and zinc; BD − P = basal diet with reduced available phosphorus (75% of the basal diet level); BD + Ca = basal diet containing 4.59% Ca, representing a 15.3% increase relative to the basal diet.

SEM: Standard error of the mean.

Values within the same column bearing different superscripts (a–e) differ significantly (P ≤ 0.05).

Dietary treatments exerted no significant influence on the frequency of damaged eggs between weeks 90 and 94 of age (P > 0.05). During weeks 94–98, the dietary treatments significantly altered the frequency of damaged eggs (P = 0.004). The highest percentages of damaged eggs were observed in the control and Mn-supplemented groups (11.82% and 11.31%, respectively), whereas all other dietary treatments resulted in significantly lower values. Similarly, a significant treatment effect on damaged egg incidence was observed over the entire experimental period (P = 0.02). The Mn-supplemented group exhibited the highest overall percentage of damaged eggs (11.74%), followed closely by the control group (11.09%), both of which were higher than the remaining treatments.

### Egg quality traits

Data demonstrating the impact of distinct mineral dietary treatments on overall egg quality traits are summarized in Table 5.The experimental diets exerted a significant effect on specific gravity values (P = 0.05). Hens fed the diet containing 30% higher Ca than the recommended requirement exhibited the highest specific gravity value (1.095), which was significantly greater than that of the control group (1.088) and Mn-supplemented treatment (1.090). The dietary regimens also exerted a significant effect on the Haugh unit, a key indicator of albumen integrity (P < 0.0001). The highest Haugh unit was recorded in hens receiving the high-Ca diet (85.74), which was statistically similar to the control group (85.07) and the Mg-supplemented treatment (84.70). In contrast, Zn supplementation resulted in the lowest Haugh unit value (78.61), followed by Mn supplementation (80.50). Both yolk weight and yolk index values remained statistically unchanged across all experimental groups (P > 0.05).

**Table 5:** Effect of different mineral supplementation on egg quality characteristics of aged laying hens (overall experimental period).

	Specific Gravity		Haugh Unit	Yolk Index	Yolk Weight (g)	Shell Weight (g)	Shell Thickness (mm)
BD		1.088ᵇ	85.06ᵃᵇ	3.09	30.14	8.86ᵇ	0.35ᵇ
BD + Mg		1.091ᵃᵇ	84.70ᵃᵇ	2.94	28.24	9.29ᵃ	0.37ᵃ
BD + Mn		1.090ᵇ	80.49ᵈ	3.24	29.29	8.86ᵇ	0.37ᵃ
BD + Zn		1.091ᵃᵇ	78.61ᵉ	3.26	28.76	8.38ᶜ	0.35ᵇ
BD + Mg + Mn + Zn		1.091ᵃᵇ	83.47ᵇᶜ	3.24	29.93	8.40ᶜ	0.36ᵃᵇ
BD - P		1.091ᵃᵇ	82.55ᶜ	3.12	27.97	8.45ᶜ	0.36ᵃᵇ
BD + Ca		1.095ᵃ	85.74ᵃ	3.10	29.10	9.04ᵇ	0.37ᵃ
SEM		0.001	0.56	0.09	0.68	0.06	0.01
P-value		0.05	<0.0001	0.17	0.23	<0.0001	0.04

BD = basal diet (control); BD + Mg = basal diet supplemented with magnesium; BD + Mn = basal diet supplemented with manganese; BD + Zn = basal diet supplemented with zinc; BD + Mg + Mn + Zn = basal diet supplemented with magnesium, manganese, and zinc; BD−P = basal diet with reduced available phosphorus (75% of the basal diet level); BD + Ca = basal diet containing 4.59% Ca, representing a 15.3% increase relative to the basal diet.

SEM: Standard error of the mean.

Values within the same column bearing different superscripts (a–e) differ significantly (P ≤ 0.05).

Dietary modifications significantly altered eggshell weight (P < 0.0001). Hens supplemented with Mg produced the heaviest eggshells (9.29 g), significantly exceeding all other treatments. The high-Ca diet also resulted in relatively high shell weight (9.04 g), whereas Zn supplementation produced the lightest shells (8.39 g). The combined mineral supplementation and reduced P treatments similarly yielded lower shell weights.

Shell thickness differed significantly among treatments (P = 0.04). The thickest shells were observed in hens fed the Mn-supplemented diet, the high-Ca diet, and the Mg-supplemented diet (0.37 mm), all of which were significantly greater than the control group and Zn supplementation groups (0.35 mm for both).

## Discussion

### Productive performance

The late laying period is characterized by declining productive performance and eggshell quality, partly due to impaired nutrient absorption, mineral metabolism, reproductive efficiency, and depletion of skeletal Ca reserves (Rezaei and Mens, 2024; Hou et al., 2025). In the present study, dietary mineral supplementation affected several performance parameters in hens aged 90–98 weeks. Magnesium and Mn supplementation increased FI, whereas combined Mg + Mn + Zn reduced FI. Although EM was unaffected, most mineral-supplemented groups showed improved EP and FCR during the late production phase. These results suggest that mineral supplementation may help sustain productivity in aged laying hens as nutrient utilization and reproductive capacity decline.

Dietary Mg supplementation increased FI and EP and improved feed conversion efficiency, broadly consistent with Skrivan et al. (2016), who evaluated diets containing 1.52–1.56 or 4.0 g Mg/kg and observed improved production traits at the higher Mg concentration. Similarly, Belkameh et al. (2021) reported increased FI and hen-day EP as dietary Mg increased from a basal 3.19 to 5.71 g/kg. In contrast, Kim et al. (2013a) increased dietary Mg from 1.6 to 2.3, 2.6, and 3.0 g/kg by adding MgO and observed no improvements in EP, EW, or FCR, along with reduced FI. Namgung et al. (2010) likewise reported no beneficial effects of MgO supplementation on FCR or EW in 95-wk-old hens.These discrepancies may reflect differences in basal and total dietary Mg concentrations, hen age, mineral source, and dietary Ca and P levels. In the present study, the basal diet contained approximately 1.60 g Mg/kg, while 4 g MgO/kg (43% purity) provided approximately 1.72 g elemental Mg/kg, resulting in a calculated total Mg concentration of 3.32 g/kg. Therefore, responses to Mg supplementation should be interpreted based on total dietary Mg rather than supplemental dose alone.

Magnesium is an established enzymatic cofactor involved in pathways regulating basic cellular metabolism and nucleic acid synthesis (Elin, 1994; Shastak and Rodehutscord, 2015). In aged hens, a baseline decline in metabolic efficiency can limit energy turnover needed for maintenance and laying performance. While metabolic parameters were not directly measured in the present 56-day trial, it is hypothesized that supplemental Mg may potentially support cellular energy fluxes and neuromuscular function, which could theoretically influence feed motivation and nutrient conversion efficiency (Shastak and Rodehutscord, 2015; Gu et al., 2021; Rezaei and Mens, 2024).

Supplementation with 50 mg MnSO_4_/kg increased FI and improved FCR but did not significantly affect EP, consistent with Lim and Paik (2003) and Zhang et al. (2017b), who reported limited effects of Mn supplementation on laying performance. In contrast, Zarghi et al. (2023) observed improved EP, EM, and FCR, but no effect on EW, in 83-wk-old hens supplemented with 30–90 mg Mn/kg. Similarly, the meta-analysis of Mbajiorgu et al. (2025) indicated that Mn generally improves productive performance. The limited response in the present study may suggest that the basal diet provided sufficient Mn to sustain performance in hens approaching the end of the laying cycle.

Supplementation with 30 mg ZnSO_4_/kg produced one of the most consistent positive responses, improving late-phase EP and FCR. This finding agrees with the meta-analysis of Ogbuewu and Mbajiorgu (2022), which reported significant improvements in EP, EW, and FCR following Zn supplementation, particularly in older hens. Similarly, Niknia et al. (2022) observed improved FI, EP, and FCR in 80-wk-old hens supplemented with 40–80 mg Zn/kg diet. The beneficial effects of Zn may be attributed to its role as a cofactor for metalloenzymes involved in nutrient metabolism, antioxidant defense, and reproductive processes (Elnesr et al., 2024). Although antioxidant and physiological markers were not measured, enhanced antioxidant protection may help alleviate age-related oxidative stress and support performance during the late production cycle (Gu et al., 2021).

Interestingly, the combined Mg + Mn + Zn treatment reduced FI relative to the individual mineral treatments while maintaining similar EP and improving FCR. This pattern could hypothetically reflect alterations in nutrient utilization efficiency under multi-element supplementation, though specific metabolic efficiency markers were not assessed in the present study. Alternatively, competitive interactions among divalent minerals during intestinal absorption may have contributed, as Mg, Mn, and Zn share intestinal transport pathways and simultaneous supplementation at elevated concentrations could potentially affect their uptake (Henry and Miles, 2000; Świątkiewicz et al., 2014).

The diet containing 25% less available P improved EP during the late laying phase and enhanced feed efficiency without adversely affecting EM. These findings are consistent with recent reports showing that older hens can maintain performance at lower NPP concentrations than those traditionally recommended. Rodehutscord et al. (2023) reported that diets containing approximately 2.2 g/kg NPP adequately supported laying performance when Ca levels ranged from 35 to 40 g/kg. Likewise, Shao et al. (2025) found that reducing available P from 0.32% to 0.27% had no negative effect on FI, EP, EM, or FCR in aged hens, and Pagnussatt et al. (2025) observed no impairment in performance when NPP was reduced to 2.2 g/kg. It is hypothesized that the observed performance responses could potentially relate to altered Ca utilization under a modified dietary Ca:P ratio (Bar et al., 2002). Notably, the P reduction applied in this trial remained above the deficiency thresholds previously associated with impaired laying performance in hens (Bar and Hurwitz, 1984; Sohail and Roland, 2002; Keshavarz, 2003).

The high-Ca treatment, providing 15% more Ca than the control diet, improved EP, FCR, and EW over the 56-day period. The positive effect of Ca on EW observed in this study agrees with Attia et al. (2020), who reported a progressive increase in EW in 60-week-old H&N Brown hens fed diets containing 3.5, 4.0, and 4.5% Ca. Similar responses were reported by Safaa et al. (2008), who found improved EP, EM, and FCR with increasing Ca intake in hens aged 58 to 73 weeks. Although some studies have reported minimal or no response to dietary Ca in hens between 40 and 80 weeks of age (Castillo et al., 2004; Cufadar et al., 2011; An et al., 2016), it is hypothesized that the positive performance response in the present trial could relate to age-associated shifts in Ca dynamics. While intestinal absorption efficiency and skeletal mobilization rate were not directly measured in these 90- to 98-week-old hens, age-related reductions in these physiological pathways (Diana et al., 2021) may potentially alter dietary Ca needs during the late production cycle.

### Egg quality traits

The most pronounced effects of mineral supplementation in the present study were observed for shell-related traits. The high-Ca diet improved specific gravity and shell thickness, Mg supplementation increased shell weight, and Mn supplementation enhanced shell thickness. Moreover, Zn supplementation, either alone or in combination with Mg and Mn, markedly reduced the incidence of damaged eggs during the later stages of production.

The positive effect of Ca on shell quality aligns with its role as the principal structural component of the eggshell, with literature suggesting that approximately 60–75% of shell Ca is derived directly from the diet and 25–40% is mobilized from medullary bone during calcification (Elnesr et al., 2024; Salehi et al., 2025). In aged hens, physiological changes such as reduced Ca absorption capacity and greater dependence on skeletal reserves can impair shell formation and increase breakage risk (Rezaei and Mens, 2024). It is hypothesized that the higher dietary Ca level may have supported shell mineralization by increasing the availability of absorbable Ca, thereby potentially altering reliance on skeletal reserves. However, Ca utilization is also influenced by Ca balance, P availability, vitamin D status, and mineral source (Taghizadeh et al., 2024; Maula et al., 2025); therefore, the observed improvements likely reflect changes in Ca supply and physiological utilization rather than dietary Ca intake alone.

The beneficial effects of Mg supplementation are consistent with previous reports. Mahdi et al. (2021) observed increases in shell weight, shell thickness, and shell strength with increasing dietary Mg concentrations, while Kim et al. (2013a, 2013b) and Seo et al. (2010) reported improvements in shell strength and thickness. Magnesium is the second most abundant mineral in eggshells, with literature indicating its involvement in crystal formation and mineral deposition through the modulation of calcite crystal morphology and shell ultrastructure (Kim et al., 2022a). Improved Mg availability may therefore enhance shell structure and mechanical resistance by promoting the nucleation and organized growth of CaCO_3_ crystals, particularly in the palisade layer, which is a major determinant of shell strength (Kim et al., 2022a).

Similarly, Mn supplementation improved shell thickness in the present study. Numerous studies have highlighted the importance of Mn in eggshell formation (Xiao et al., 2014; Zhang et al., 2017b; Cui et al., 2019), and a recent meta-analysis by Mbajiorgu et al. (2025) confirmed that dietary Mn supplementation significantly increases shell thickness. Literature suggests that Mn participates in the synthesis of proteoglycans and glycosaminoglycans within the organic shell matrix and may modulate genes associated with shell gland function and mammillary-knob formation (Zhang et al., 2018). Although matrix biochemistry, gene expression, and shell ultrastructure were not directly evaluated in this 56-day experiment, it is hypothesized that these potential physiological pathways could theoretically support shell matrix organization and contribute to the observed improvements in shell thickness.

A major finding of the present study was the marked reduction in damaged eggs following mineral supplementation, particularly in hens receiving Zn alone or in combination with Mg and Mn. Eggshell defects and breakage are major economic concerns in aged laying hens because shell quality declines progressively with age(Wistedt et al., 2019). Thus, the lower incidence of damaged eggs in the supplemented groups indicates that targeted mineral nutrition may help preserve eggshell integrity during late lay. The literature regarding the biological roles of Zn provides a plausible basis for this response. Zinc serves as a cofactor for carbonic anhydrase, which catalyzes the hydration of CO_2_ to HCO_3_^-^, supplying carbonate for CaCO_3_ deposition during shell mineralization. Additionally, Zn is involved in the synthesis of organic shell matrix proteins that influence crystal nucleation, growth, and orientation (Zhang et al., 2017a; Elnesr et al., 2024). However, previous studies have reported inconsistent effects of Zn on egg breakage. Qiqi et al. (2020) found no significant effect of Zn supplementation on broken egg rate, whereas Kim et al. (2022b) reported lower rates of broken and shell-less eggs in hens fed mineral premixes containing Zn, Mn, Mg, and other trace minerals compared with birds on a basal diet. Improvements in shell quality following Zn supplementation have also been reported by Soroush et al. (2015)and AlirezaeiShahraki et al. (2024), while the meta-analysis by Ogbuewu and Mbajiorgu (2022) showed a consistent positive effect on eggshell thickness. These differences may be explained by variation in hen age, mineral source and dose, dietary composition, environmental conditions, and supplementation duration.

The greater reduction in damaged eggs in the combined mineral groups suggests that the response may reflect complementary interactions among Zn, Mg, and Mn rather than an effect of Zn alone (Henry and Miles, 2000). These trace minerals may act synergistically by supporting distinct yet interconnected processes involved in shell formation. Their combined supplementation may therefore improve the efficiency of shell mineralization and matrix organization, ultimately enhancing shell integrity(Henry and Miles, 2000; Shastak and Rodehutscord, 2015; Elnesr et al., 2024). Such complementary effects may be particularly beneficial in older hens, in which declining Ca utilization and shell-forming efficiency increase susceptibility to shell defects and breakage.

In contrast to shell traits, dietary mineral supplementation had limited effects on internal egg quality. The high-Ca diet resulted in the highest Haugh unit values, indicating improved albumen quality, whereas Zn supplementation decreased Haugh units. Yolk weight and yolk index were unaffected, suggesting that mineral supplementation primarily influenced eggshell formation rather than yolk deposition. The limited response of Haugh unit to Mg supplementation is consistent with previous studies reporting no significant effect on albumen quality (Namgung et al., 2010; Sedgh-Gooya et al., 2018; Kim et al., 2013a). Likewise, Zarghi et al. (2023) reported no effect of Mn supplementation on Haugh unit or other internal egg quality traits. In contrast, some studies have reported improved Haugh unit values with Zn supplementation (Soroush et al., 2015), and the meta-analysis by Ogbuewu and Mbajiorgu (2022) found an overall positive effect of Zn on Haugh unit. The inconsistent responses among studies likely reflect differences in hen age, mineral source, supplementation level, and experimental conditions.

## Conclusions

The present study indicates that mineral supplementation can influence productive performance and eggshell quality in laying hens during the late production period (90–98 weeks). Supplementation with Mg, Mn, and Zn improved selected production and shell-quality responses, while the combined Mg + Mn + Zn treatment reduced FI without adversely affecting EP. In addition, reducing available P by 25% did not impair performance and improved feed efficiency, whereas increasing dietary Ca by 15% enhanced EP, EW, and shell quality. Collectively, these findings indicate that maintaining an appropriate balance of Ca, P, Mg, Mn, and Zn is important for sustaining productivity and eggshell quality in aged laying hens.

Further research is needed to extend the experimental period to later stages of lay, with comprehensive analysis of experimental diets to verify actual mineral concentrations. Future studies should also evaluate different mineral sources and inclusion levels, as well as Ca–P–trace mineral interactions under environmental challenges such as heat stress, to better understand their effects on mineral utilization, eggshell quality, and skeletal integrity in aged laying hens.

## Author contributions

Shahab Pourlorestani: 1. Substantial Contributions: The acquisition, analysis, or interpretation of data. 2. Drafting or Reviewing: Writing the manuscript draft 3. Final Approval: Reviewing and explicitly approving the exact, final version of the manuscript before it goes to press. 4. Accountability: Agreeing to be personally and publicly responsible for all aspects of the work, ensuring that any questions regarding the accuracy or integrity of the research are thoroughly investigated and resolved. Mehran Torki: 1. Substantial Contributions: Substantive input into the conception or design of the work, and the interpretation of data. 2. Drafting or Reviewing: Reviewing it critically for crucial intellectual content. 3. Final Approval: Reviewing and explicitly approving the exact, final version of the manuscript before it goes to press. 4. Accountability: Agreeing to be personally and publicly responsible for all aspects of the work, ensuring that any questions regarding the accuracy or integrity of the research are thoroughly investigated and resolved. Reza Mahdavi: 1. Substantial Contributions: Substantive input into the conception or design of the work, and the interpretation of data. 2. Drafting or Reviewing: Reviewing it critically for crucial intellectual content. 3. Final Approval: Reviewing and explicitly approving the exact, final version of the manuscript before it goes to press. 4. Accountability: Agreeing to be personally and publicly responsible for all aspects of the work, ensuring that any questions regarding the accuracy or integrity of the research are thoroughly investigated and resolved.

## Ethics declaration

### Animal subject

This study was conducted in accordance with the ARRIVE (Animal Research: Reporting of In Vivo Experiments) guidelines. This study was approved by the Animal Ethics Committee of Razi University. (Approval No. IR.RAZI.AEC.1400.003).

## Disclosures

The authors declare that they have no known competing financial interests or personal relationships that could have appeared to influence the work reported in this paper.
